# Injectable Cryogels in Biomedicine

**DOI:** 10.3390/gels7020038

**Published:** 2021-04-01

**Authors:** Duygu Çimen, Merve Asena Özbek, Nilay Bereli, Bo Mattiasson, Adil Denizli

**Affiliations:** 1 Department of Chemistry, Hacettepe University, Ankara 06800, Turkey; duygucimen@hacettepe.edu.tr (D.Ç.); asenaozbek@hacettepe.edu.tr (M.A.Ö.); bereli@hacettepe.edu.tr (N.B.); 2 Department of Biotechnology, Lund University, Box 124, 221 00 Lund, Sweden; bo.mattiasson@biotek.lu.se

**Keywords:** injectable cryogel, supermacroporous, polymer, biomedicine application

## Abstract

Cryogels are interconnected macroporous materials that are synthesized from a monomer solution at sub-zero temperatures. Cryogels, which are used in various applications in many research areas, are frequently used in biomedicine applications due to their excellent properties, such as biocompatibility, physical resistance and sensitivity. Cryogels can also be prepared in powder, column, bead, sphere, membrane, monolithic, and injectable forms. In this review, various examples of recent developments in biomedical applications of injectable cryogels, which are currently scarce in the literature, made from synthetic and natural polymers are discussed. In the present review, several biomedical applications of injectable cryogels, such as tissue engineering, drug delivery, therapeutic, therapy, cell transplantation, and immunotherapy, are emphasized. Moreover, it aims to provide a different perspective on the studies to be conducted on injectable cryogels, which are newly emerging trend.

## 1. Introduction

Nowadays, the need for injectable biomaterials that have hemostatic effects in the field of tissue engineering and that are used in tissue regeneration is increasing. Furthermore, these biomaterials are preferred in order to minimize the risks and complications that may occur with surgical implantation. In addition to being biocompatible, three-dimensional (3D) scaffold biomaterials with physicochemical properties such as hardness, elasticity, and biological degradation provide a structural support and physical environment for cell attachment and subsequent tissue development. Cryogels, one of the biomaterials with injectable 3D scaffold structures, can be loaded with therapeutic agents or cells in line with the needs developing in clinical and tissue engineering studies. Thanks to their highly macroporous and interconnected structures, cryogels provide a suitable microenvironment for cell transmission, cellular infiltration, and neovascularization [1]. In recent years, the most widely used biocompatible polymeric biomaterials in biomedicine applications are cryogels.

Cryotropic gelation is a gel formation process that is used to produce supermacroporous hydrophilic gels called cryogels. Cryogels are interconnected supermacroporous materials that can be synthesized at sub-zero temperatures using an existing activator/initiator pair [2,3]. Polymerization occurs in a frozen monomer solution in the interstitial spaces between ice crystals. Ice crystals act as a porogen throughout the scaffold during polymerization, and a supermacroporous polymer is formed after thawing [4,5]. Cryogels have important properties such as flexibility, large pores (10–200 μm), high mechanical strength, short diffusion paths, and good biocompatibility [6,7]. Cryogels can be synthesized in different forms such as powder, column, bead, sphere, membrane, monolithic, and injectable. Injectable cryogels with 3D porous structures are used for minimally invasive implantation in biomedicine applications, thanks to the advantages provided by their elastic structures, as well as their adjustable micron-scale and desired geometric structures. Injectable cryogels can be used in a variety of biomedicine applications, including diagnosis, therapy, drug delivery, and tissue engineering [8,9,10].

The purpose of this review is to explain the properties of injectable cryogels and their use in biomedicine applications, to sample literature studies about these uses, and to gain a different perspective for new studies. Firstly, the preparation of cryogels and their structural properties have been explained, with a focus on injectable cryogels obtained by synthesizing cryogels in micron-scales. In the following sections, injectable cryogels are discussed in regard to different subjects such as wound healing, hemostasis, antibacterial activity, tissue regeneration, and cell and drug delivery, and the uses of these biomaterials in biomedical fields are summarized. It is thought that this review will be a guide for the design of injectable cryogels, improving their structural properties and making their use more common in different biomedical fields for future studies.

## 2. Cryogels

**Figure 1:**
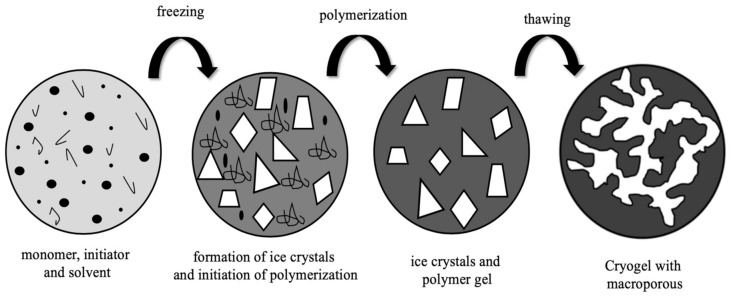
Illustration of supermacroporous cryogel preparation.

Cryogels are interconnected supermacroporous materials that can be synthesized at sub-zero temperatures by using a monomer solution and an available initiator/activator pair [11,12]. At a temperature below the freezing point of the solvent, the polymerization or crosslinking reaction occurs in the semi-solid phase containing the monomer [13,14]. After the reaction product melts at room temperature, a loose macroporous cryogenic gel is prepared and referred to as a cryogel (Figure 1). Water is used as a solvent, dispersant, and porogen. Water helps the cryogel to form its macroporous structure with a phase separation in which the frozen ice crystals of water temporarily transform into a fully interconnected cryogenic skeleton [15,16].

Cryogels are produced with a wide variety of morphologies and properties that can be used for different applications. Cryogels are heterogeneous, non-transparent, large macroporous materials [17,18]. The macroporous size of cryogels varies depending on experimental synthesis conditions such as the concentrations and ratios of monomer, solvent, initiator and cross-linkers, temperature, and freezing rate. Supermacroporous cryogels are usually spongy materials with interconnected porous structures. The properties of cryogels depend on the pore structure, the thickness of the pore wall, and the density expressed as polymer density in the swollen pore walls [19,20].

The pore size of the cryogel is mostly dependent on many factors such as the initial concentration of monomers, physicochemical properties, and the degree of freezing. Additionally, it controls the mass transfer in the cryogel together with the connection between the pore networks. The macroscopic mechanical properties of the cryogel are determined by its pore wall thickness and density. Cryogels contain large amounts of liquid. The most common solvent used is water [21].

The highly porous interconnected structure allows for the unhindered diffusion of all solutes, including macromolecules and even colloid particles (protein micelles and viruses) in the cryogels with pores larger than 50 µm. This property makes cryogels attractive materials for bioseparation and cell culture [22,23,24,25]. The synthesis, structure, and applicability of cryogels that can be used in a variety of biomedicine applications, including enzyme activity [26], drug delivery [27], protein purification [28], and tissue engineering [29], are very important. The heat and mass transfer of cryogels is faster than normal gels with the same chemical composition. Moreover, the response time of cryogels is fast at short distances in the thin walls of macroporous structures [30,31,32,33]. The flow channel in a cryogel is large enough due to the interconnected macropore structure. Pore wall thickness and density determine the macroscopic mechanical properties of the cryogel, and the interconnectedness of the pores in the cryogel controls mass transport [34,35,36,37].

Cryogels, which are spongy materials prepared by the cryopolymerization of an aqueous solution containing gel-forming monomers under freezing conditions, can be prepared in the form of powder, column, bead, sphere, membrane, monolithic, and injectable material. Cryogels have advantages such as high blood compatibility, high water content, resistance to degradation, no toxicity, and pressure drop properties. These advantages allow them to be used without any diffusion problems when working with biological macromolecules [38,39,40,41,42].

## 3. Injectable Cryogels

In recent years, injectable cryogels have been investigated for the minimally invasive implantation of 3D skeletons. The injectability of cryogels is a result of their elastic structure. These properties are very important for applications in soft tissue reconstruction when cryogels are injected subcutaneously. Cryogels have unique structural properties such as shape memory properties, degrees and mechanisms of crosslinking, interconnected macropores, and dense polymer walls [43].

Both micro- and macroscale injectable cryogels are used to provide a 3D porous structure, help protect encapsulated biological agents against degradation, and control the delivery of mammalian cells and biomolecules to host tissues. In addition, particle-based biomaterials, with their small size, can show insufficient retention at the injection site. This limitation increases the need for repeated injections, causing both serious side effects and increased costs. To reduce these limitations, macroscale cryogels are being investigated, as they can form a localized structure at the injection site [44,45].

With their shape memory properties and sponge-like macroporous structural features, injectable cryogels, which have similar properties to cryogels, can be easily recognized and accepted by the body with cell and purposeful ligand immobilization. For this reason, they have the potential to be the preferred biomaterial for many biomedical applications. These biomaterials have been used in many biomedicine applications such as immunotherapy, drug delivery, and tissue engineering. [27,46,47,48,49,50].

Biodegradable cryogels that provide temperature sensitive sol–gel transitions between body and room temperature are used in biomedicine applications. Biodegradation, which is an important element when preparing scaffolds for tissue engineering, is important for facilitating new tissue formation and integration upon implantation [51,52,53,54,55].

Macro-scale protein drug delivery systems are used to control the delivery of drugs to specific tissues. Careful material selection is often required for establishing an effective polymer drug delivery system for a particular protein, achieving the desired protein release profile, and maintaining bioactivity [56]. In drug-loaded cryogels, because the porous structure triggers the burst release of the drug, the use of a nanocarrier to overcome this situation could be a potential candidate for both controlling the drug release rate and enhancing the cryogels [57].

For many biomedicine applications, there is an increasing need for the tissue engineering of advanced 3D scaffolds to provide mechanical and structural support to tissue and cell regeneration. For this reason, 3D scaffolds should be made of resorbable and biocompatible polymers and have large macropores linked to each other [58].

Bone defects often require invasive surgery and are difficult to treat. Injectable cryogels are often used for bone tissue regeneration in the treatment of pathological fractures. Hixon et al. used injectable alginate-based cryogels with platelet-rich plasma (PRP). Although the use of PRP adversely affected the physical properties of cryogels, the freeze–thaw cycle improved their porosity and compressibility. These PRP-loaded, injectable cryogels have been shown to increase the proliferation and mineralization of human bone osteosarcoma cells. Injectable cryogel studies in biomedicine will be discussed in the following sections [59].

Improving the properties of injectable cryogels has provided promising results for biomedicine applications, and many methods are currently being studied in this regard. One of the major problems is insufficient control over the release of biomolecules, particularly low molecular weight components. Due to the high porosity in their structure, drug-loaded cryogels result in a burst release that limits their potential as a drug delivery carrier. Koshy et al. used injectable cryogels to improve drug release kinetics. Injectable cryogels combined with laponite nanoparticles (NPs) preloaded with immunomodulatory factors. Unlike freely injectable laponite cryogels, laponite NPs immobilized in injectable cryogels have been observed to inhibit burst release. In addition, the varying laponite content better adjusts the release kinetics from the cryogels while maintaining the injectability of the syringe [60].

Cryogels are reversible, collapsible, and elastic materials, and these properties make them suitable for injection. Generally, 16-gauge (16 G) is the preferred needle size for injecting 8 × 8 × 1 mm³. 16 G needles reduce penetration compared to surgical implantations, so the use of smaller needles will also reduce tissue damage and eliminate this problem. Therefore, optimization studies are required to improve the injectability of cryogels. While this size of injectable cryogel is used in biomedicine applications where large scaffolds are required, some optimization studies are carried out to avoid problems. In addition to the geometry and crosslinking mechanisms of injectable cryogels, it is necessary to optimize macrostructural properties such as pore size, pore connectivity, and flexibility. It is important to design injectable cryogels to prevent postoperative complications [10,61].

## 4. Injectable Cryogels in Biomedicine Applications

### 4.1. Wound Repair Applications of Injectable Cryogels in Tissue Engineering

Different skin defects appear due to diseases, various traumas, aging, or post-operative situations. In most cases, the spontaneous healing of these wounds takes time, and the individual faces the risk of infection that may occur in the injured area. At this point, studies in the field of tissue engineering and biocompatible, biodegradable materials developed in this field gain great importance. One of them is injectable cryogels, whose high mechanical and physical stability contribute to tissue regeneration. Another superior feature of injectable cryogels is their placement with minimal intervention in the tissue to avoid complications such as wound infection due to surgical scaffold implantation. In addition, they support the treatment with the antimicrobial effects of the materials used in injectable cryogel synthesis [62,63,64].

Sener et al. aimed to prepare injectable zwitterionic cryogels that help speed up the wound healing process. Skin wounds from various causes, such as diabetes, burns, vascular insufficiency, and post-surgical wounds, are difficult to heal and take a long time. This situation increases the need for materials that will contribute to the repair of the injured area. For this purpose, firstly, they synthesized crosslinker-free injectable cryogels using different monomer compositions such as 2-hydroxyethyl methacrylate (HEMA), 3-[[2-(methacryloyloxy)ethyl] dimethylammonio] propionate (CBMA), or [2-(methacryloyloxy)ethyl]dimethyl-(3-sulfopropyl) ammonium hydroxide (SBMA) and different copolymer molar ratios, and then loaded miRNA146a coupled cerium oxide nanoparticles into these cryogels. As a result of experiments with biocompatible cryogels with advanced mechanical properties in a diabetic mouse model, it was proven that the wound healing time was accelerated, and the risk of infection was also reduced [65].

Zhao et al. prepared injectable antibacterial conductive nanocomposite cryogels for use in the wound healing process and as a hemostatic agent. A large number of people are faced with death around the world, especially in emergencies due to the inability to control hemorrhage. Therefore, the use of hemostatic agents to control bleeding quickly and effectively is critical. For this, they designed glycidyl methacrylate (GMA) functionalized quaternized chitosan (QCSG)-based cryogels reinforced with carbon nanotubes (CNTs). The hemostatic effects of shape memory cryogels obtained in this way, which have strong mechanical properties, were studied. As a result of their work with mouse and rabbit models, it was observed that the cryogels they prepared performed better than the gelatin hemostatic sponge and gauze [66].

Çetin et al. demonstrated the preparation of micron-scale cryogels with antibacterial properties, called microcryogels. Lysozyme, which is considered an antibacterial agent of our body against antibiotic-resistant bacteria, also has an important place in the field of biotechnology because of its antineoplastic effect. Based on this information, three different types of gelatin-based microcryogels were obtained by loading lysozyme solutions at different concentrations. Microcryogels were characterized in detail in terms of gelation efficiency, surface area, thermal stability, surface morphology, degree of swelling, and macroporosity. Thanks to the MTT method, it was possible to evaluate the cell viability, and a negligible reduction was observed for a period of 72 h. The prepared microcryogels greatly impacted the growth of *E. coli*, *B. subtilis*, and *S. aureus*, thus the antibacterial activity of microcryogels was proven with the large contribution of lysozyme [67].

In cases of incompressible bleeding in deep wounds, the production of agents with high hemostasis is still a challenge. Zhao et al. presented antioxidant and biodegradable injectable dry cryogels with a distinguished photo-thermal capacity to be able to control bleeding. In the synthesis of cryogels, polydopamine cross-linked chitosan was used because chitosan posesses antibacterial activity as well as good hemostatic effects. Dopamine concentrations of the cryogels were studied at values ranging from 0.5 to 9.0 mg/mL to determine the optimum contents. As a result of detailed animal model experiments, where statistical analyses were also carried out, it was revealed that the CS20/PDA4.5 coded cryogel, consisting of 4.5 mg dopamine and 20 mg chitosan, was a much better hemostatic candidate compared to gelatin sponge and combat gauze [68].

**Figure 2:**
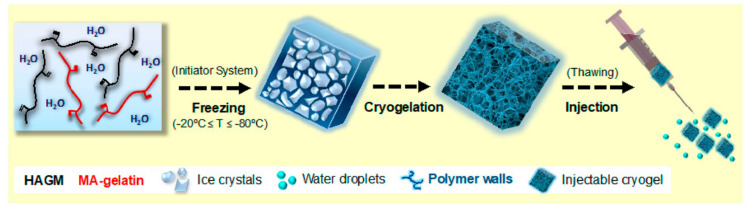
Schematic representation of the entire process involved in forming injectable cryogels. The methacrylate derivatives of HA (HAGM) and/or gelatin (MA-gelatin) are dissolved in water containing an initiator system. Then, it is frozen at three different temperatures: −20, −50 and −80 °C. Cryogelation occurs around the ice crystals. Interconnected spaces are obtained after melting at room temperature. Ultimately, macroporous cryogels can be injected successfully with a syringe through a 16 G hypodermic needle. [51].

Besides being an extracellular matrix (ECM) component, hyaluronic acid (HA) is a polysaccharide that it has an important role in conditions such as wound healing and angiogenesis. In the studies of cryogel scaffolds related to this, it was observed that the structure formed with HA has various properties such as good flexibility and rapid absorption of body fluids, which are important in the field of tissue engineering. In addition, the combination of HA with gelatin from hydrolyzed collagen, which is abundant in our body, has been used to improve cell adhesive characteristics. Rezaeeyazdi et al. fabricated injectable hyaluronic acid-co-gelatin cryogels for use in tissue engineering (Figure 2). In the study, important points such as pore size, swelling rate, syringe injectability, and mechanical behavior of cryogels synthesized under different conditions including fabrication temperature were emphasized. It was stated that these biomaterials, which demonstrated advance cell adhesive properties with minimal cytotoxicity, were promising due to their contribution to the field of new, minimally invasive treatments and tissue regeneration [51].

**Figure 3:**
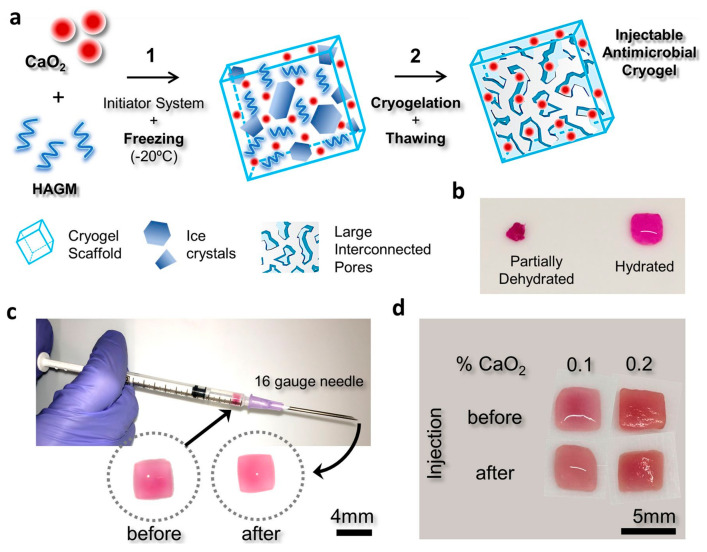
Schematic representation of the entire process involved in forming injectable cryogels: (**a**) the cryogelation procedure of microcomposite cryogels containing CP: (1) Cryogels were manufactured using 4% HAGM with different amounts of CP (0–0.2% CaO_2_); an initiator system is added to an aqueous HAGM solution, and then cryopolymerization is performed at −20 °C. (2) Ice crystal formation and gelation occurs. With the thawing of the ice crystals, an interconnected macroporous cryogel is formed. (**b**) dehydrated and hydrated forms of the obtained cryogels, (**c**) pre- and post-injection cryogel forms from a 16 G hypodermic needle, and (**d**) cryogels conserve encapsulated CP after injection [69].

Navare et al. presented a study on injectable microcomposite cryogels with superior properties due to their antimicrobial identity. One of the main studies in the field of tissue engineering is the work on producing 3D scaffolds for damaged tissue repair. For this purpose, microcomposite cryogels were obtained by using methacrylated hyaluronic acid (HAGM) and calcium peroxide (CP) at different concentrations (0–0.2% CaO_2_). The principle here was that the decomposition of CP to form hydrogen peroxide and calcium hydroxide can have an antibacterial, antiviral, and antifungal effect. When studying antibiotic-resistant *Pseudomonas aeruginosa* and *Staphylococcus aureus* (MRSA) bacteria to test antimicrobiality, it was observed that the prepared cryogels inhibited these bacteria. In the mouse model experiment, a negligible inflammatory response was observed as a result of subcutaneous injections with antimicrobial cryogels after deliberate contamination with pathogenic bacteria (Figure 3) [69].

Larsson et al. reported a novel study on the synthesis of thermoresponsive cryogels with improved mechanical properties. Cellulose nanocrystals (CNCs) are known to contribute to strong durability and high elasticity in studies carried out with cryogels. As in the classical cryogelation procedure, N,N’-methylenebisacrylamide (MBAm) was used as the crosslinker to synthesize poly(N-isopropylacrylamide) (PNIPAAm) cryogels, defined as a thermoresponsive polymer. At this stage, different concentrations of acrylate-functional cellulose nanocrystals (CNC-AA) and HCl–cellulose nanocrystals (CNC-HCl) were added to the solution, and cryogels containing two different types of cellulose nanocrystals were obtained. Characterization studies with Fourier transform infrared spectroscopy (FT-IR), thermogravimetric analysis (TGA), and field emission scanning electron microscopy (FE-SEM) proved that the addition of CNCs to the cryogel structure both changed the morphological structure and contributed to the improvement of the mechanical properties. PNIPAAm_25_CNC-AA_1%_ cryogel (containing 1% (wt%) CNC-AA and ratio of MBAm: NIPAAm is 1:25] was determined as the most suitable thermoresponsive cryogel with the desired properties [70].

In the production of scaffolds that are useful to support cell proliferation in tissue damaged due to various reasons, care should be taken against situations that may pose an infection risk. Demir et al. published an experimental research on developing injectable cryogel microspheres that can be used in the field of tissue engineering. By an in situ chemical reduction method, chitosan-based cryogel microspheres containing silver nanoparticles (AgNPs) were prepared. Finally, with the preparation of silver nitrate (AgNO_3_) solution in three different concentrations, microspheres containing different proportions of AgNPs were obtained. Characterization processes with a wide variety of instruments proved that the desired microspheres were successfully synthesized. DPPH procedure is a radical scavenging of 1,1-diphenyl-2-picrylhydrazyl (DPPH) stable at 25 °C which, when prepared in methanol, exhibits a violet color. When free radical scavenging activities were evaluated by DPPH procedure, it was seen that microspheres containing AgNPs with the highest concentration took the first place. It was also stated that the same chitosan microspheres showed a high rate of antimicrobial activity against seven different microorganisms [71].

### 4.2. Cell and Drug Delivery Applications of Injectable Cryogels in Tissue Engineering

Systematic and controlled delivery of drugs used to treat diseases is valuable in terms of obtaining an efficient therapeutic effect. Otherwise, interactions of drugs delivered to non-target areas may adversely affect patients’ health and cause unwelcome complications. Thanks to the 3D polymeric scaffolds provided by tissue engineering, it is possible to perform high drug loading and to repair damaged tissue by administering the appropriate drug dose at the desired time interval. Likewise, efficiency is increased in the cell-based therapies with injectable cryogels referred to above. Injectable cryogels allow for local delivery without cells being damaged during injection [72,73,74].

Schirmer et al. conducted a study on injectable cryogels that enable the delivery of interleukin-13 (IL-13) to the brain. The application approaches that ensure the safe sustained release of protein therapeutics are still limited. Here, microscale cryogels (microcarriers) containing heparin and PEG were employed, and characterization processes were carried out. Immunomodulatory activity of IL-13 loaded microcarriers was evaluated in vitro on bone marrow-derived macrophages and in vivo in mouse brain, and it was emphasized that a slow and sustained IL-13 release was achieved with microcryogels for at least seven days [56].

The methods used in intervertebral disc degeneration (IVDD), whose treatment starts with painkillers and requires invasive operations in later stages, cannot fully provide the increase of intervertebral disc functionality. Zeng et al. manufactured injectable microcryogels to form 3D microcellular niches for use in IVDD. In research, after the encapsulation of the mesenchymal stromal cells (MSCs) in the alginate precursor, they were loaded into poly(ethylene glycol) diacrylate (PEGDA) microcryogels (PMs). The PMs-enhanced alginate hydrogel outperformed the alginate hydrogel alone in terms of protecting encapsulated cells as well as elasticity. It was concluded that microcryogels prevent cell leakage due to high pressure in an ex vivo organ culture model, and degeneration was reduced after six months in an in vivo canine IVDD model [75].

Kim et al. proposed a heparin/gelatin-based injectable cryogel that is effective in neovascularization. Injectable biocompatible materials, which are frequently preferred in minimally invasive surgery, should fully contribute to tissue regeneration. Targeted injectable cryogels were obtained as a result of different concentration ratios of heparin and gelatin. These cryogels were characterized in many aspects such as injectability, mechanical properties, swelling rate analysis, and porosity. As a result of the studies, it was observed that gelatin_1_ cryogels (containing 1% (*w*/*v*) gelatin] had a larger pore area and a higher swelling rate compared to gelatin_1.5_ cryogels [containing 1.5% (*w*/*v*) gelatin]. Considering the controlled vascular endothelial growth factor (VEGF) release of gelatin1/heparin0_.3_ cryogel (containing 1% (*w*/*v*) gelatin and 0.5% (*w*/*v*) heparin] for up to 21 days and its application in an in vivo hind limb ischemic mouse model, it was concluded that this cryogel could be used for neovascularization-promoting cell and protein delivery [45].

In Parkinson’s disease, which is a neurodegenerative disease that affects millions of people worldwide, treatment success varies depending on individual factors, treatment methods, stage of the disease, and some unknown elements. Filippova et al. designed injectable macroporous cryogels to improve cell therapy for use in Parkinson’s disease. They aimed to achieve in vivo transplantation of mature dopaminergic neurons using injectable cryogels. They performed the synthesis of injectable porous cryogel structures, called “neurothreads”, by crosslinking carboxymethylcellulose at subzero temperatures. In the study, the selection of adhesion molecules for cell seeding and in situ maturation and optimization of the functionality of cryogels was provided. The highest neural spread was determined in laminin 111 and Matrigel functionalized cryogels when covalent immobilization of the three commonly preferred neural adhesion molecules (laminin 111, collagen IV and fibronectin), as well as the extracellular matrix extract Matrigel, was performed. When the injectability of the obtained cryogels was tested, it was seen that they were resistant to compression during minimally invasive injection, and they kept mature neurons alive [76].

Bruns et al. conducted a study in which they aimed to control PEG cryogel microstructure without changing the cryogel composition. The modification process after cryogelation is problematic due to the absence of functional groups on the PEG polymer chains. The key is to ensure that PEG cryogels are pre-functionalized. Prior to cryogelation, PEG polymers were covalently pre-functionalized with the cell adhesive ligand arginine–glycine–aspartic acid–serine (RGDS). After the characterization processes, studies were carried out to prove that different polymer concentrations, polymer solvent, and quenching affect the structural properties of the cryogel. On the seventh day in cell viability analyses, cryogels containing RGDS displayed a higher rate of cell viability than cryogels without RGDS [77].

In their experimental research, Koshy et al. proposed a strategy that allows proteins to be released in a continuous and slow way. The realization of a polymer drug delivery system that can be efficient for the target protein is directly related to the various optimization processes, as well as maintaining the bioefficacy of the protein. The first step in creating injectable cryogels was the bio-orthogonal crosslinking of alginate by utilizing a tetrazine–norbornene conjugation. Proteins were pre-adsorbed onto charged laponite nanoparticles placed within the walls of the cryogels, which bind to themselves quite strongly. In detailed research, it was concluded that protein release from these nanocomposite cryogels can be controlled when different laponite concentrations were used [60].

**Figure 4:**
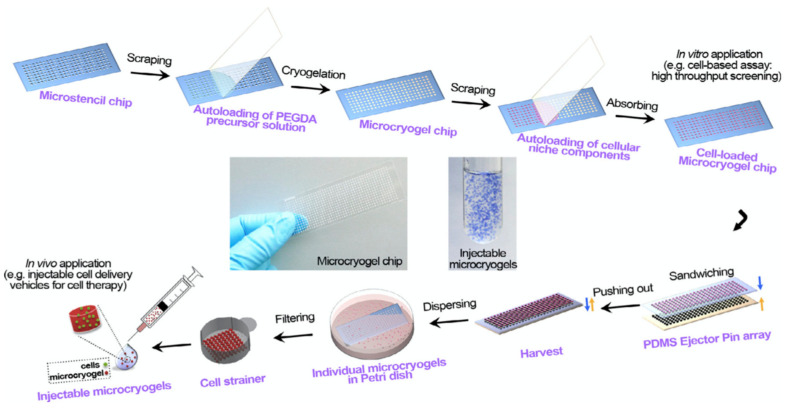
Schematic description of the preparation steps of injectable PEGDA-based microcryogels with microstencil array chips (Sandwiching step: Microcryogels are harvested by a simple push-out method using a poly(dimethylsiloxane) (PDMS) ejector pin array.) [78].

Liu et al. reported a study of injectable microscale macroporous cryogels in order for use in cell delivery. The desired success of cell therapy preferred in the field of tissue repair and regeneration depends on the availability of effective cell delivery techniques. To obtain microcryogels with pre-determined dimensions, the microstencil array chip was first designed, then PEGDA microcryogels were synthesized. In the next step, the 3D cellular niches were homogeneously loaded into injectable microcryogels (Figure 4). In the mouse model, induction of angiogenesis was observed in the relevant region after 2 weeks, thanks to injectable microcryogels with superior properties such as shape memory and high elasticity [78].

In a similar study, Zeng et al. prepared gelatin-based injectable microcryogels to contribute to the healing of skin defects. Recently, targeted and minimally invasive cell therapy has become possible thanks to injectable microcryogels that can preserve the integrity and proliferation of the cells they carry, and induce the secretion of bioactive factors. Polymethyl methacrylate (PMMA)-based microstencil array chips with special dimensions were used in the production of gelatin microcryogels. Human adipose-derived stem cells (hASCs) were then loaded into injectable gelatin-based microcryogels. To evaluate the in vivo results, the biological changes of hASCs loaded in gelatin microcryogels were compared with a conventional two-dimensional (2D) cell culture by utilizing many methods such as ELISA, bioluminescence imaging, quantitative reverse transcription-polymerase chain reaction (QRT-PCR), and Western blot analyses. In the injured mouse model experiment, it was observed that cell delivery with the obtained injectable gelatin microcryogels greatly accelerated wound healing [79].

**Figure 5:**
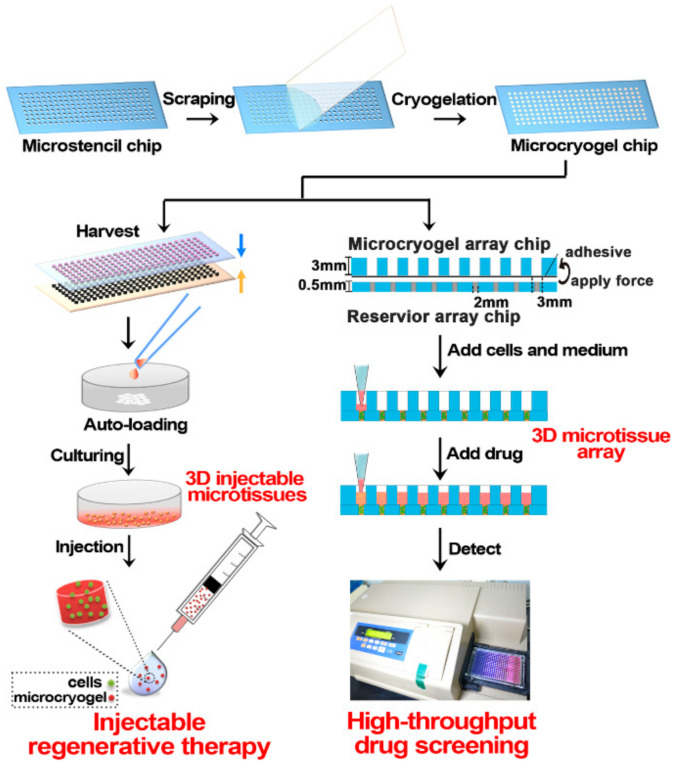
Schematic representation of 3D microtissue fabrication in regenerative treatment and drug screening. The obtained microcryogels can be auto-loaded with cells and cultured to generate 3D microtissues for injectable regenerative therapy. The other application is loading cells and culture into 3D microtissue arrays for high-throughput drug screening [80].

Li et al. designed macroporous microscale cryogels that can be loaded with various cell types to generate 3D microtissues. As well as cell survival after in vivo vaccination being an important consideration in regenerative therapy, efficient drug screening is also crucial to processing thousands of compounds at once. In this study, gelatin, which is biocompatible, cost-effective, and degradable, was used in microcryogel synthesis. The porous structure of microcryogels synthesized with PMMA-based microstencil array chips was examined by scanning electron microscope (SEM), and injectability was also tested. Then, human adipose-derived mesenchymal stromal cells were loaded into microcryogels to create 3D microtissues (Figure 5). As a result of the experimental studies, the therapeutic effect and improved cell viability were observed in the mouse limb ischemia model [80].

### 4.3. Other Applications of Injectable Cryogels

**Figure 6:**
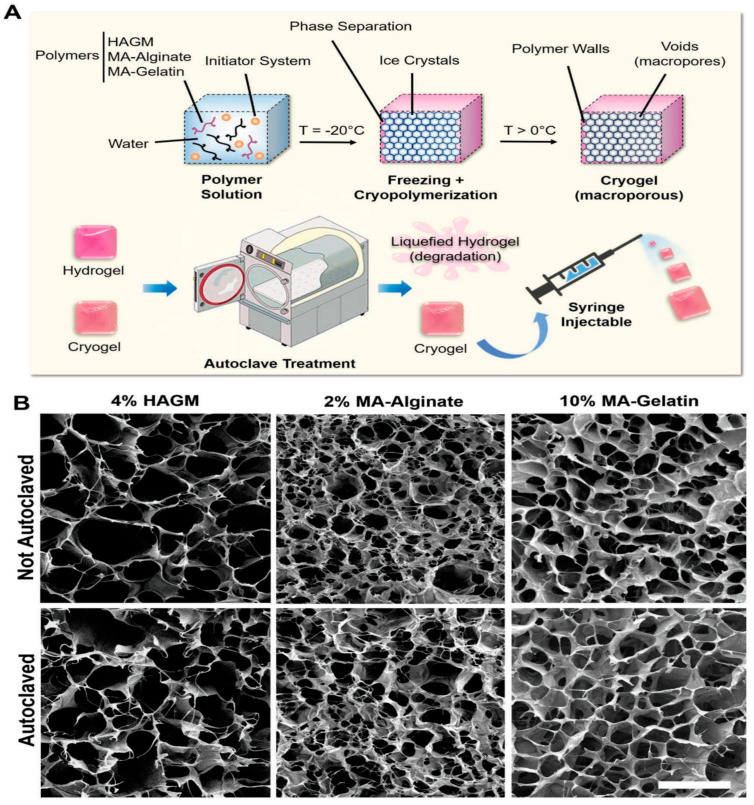
Developing different types of injectable cryogels with different polymers and autoclaving process. (**A**) Stages of the preparation process of cryogels combined with autoclaving sterilization. (**B**) SEM images of HAGM, MA-alginate and MA-gelatin cryogels (scale bar = 100 µm) [81].

Sterilization and disinfection are terms that are different from each other, and sterilization is a procedure that should be applied to all biomaterials, devices, tools, and equipment to be used clinically. Although there are a wide variety of sterilization approaches, problems such as toxicity, denaturation, and structural changes occur in these methods. At this point, the autoclave process emerges as a low-cost and reliable approach. Villard et al. demonstrated the design of different types of injectable cryogels that highlight the importance of autoclave sterilization. In the study, the production of methacrylated derivatives of hyaluronic acid (HAGM), alginate (MA-alginate), and gelatin (MA-gelatin) cryogels was realized. Structural properties of cryogels after the application of various sterilization techniques such as ultraviolet irradiation, ethanol treatment, and autoclaving were compared to their conventional hydrogel counterparts (Figure 6a). As a result of SEM analysis, it was determined that the autoclave technique had the best performance in sterilization, and unlike hydrogels, no difference was found in the important properties of cryogels such as morphological structure, mechanical stability, and injectability after autoclaving (Figure 6b). Finally, in vivo and in vitro studies of autoclaved cryogel scaffolds were evaluated, and it was observed that the biocompatibility and bioactivity properties of the relevant cryogels were preserved [81].

**Figure 7:**
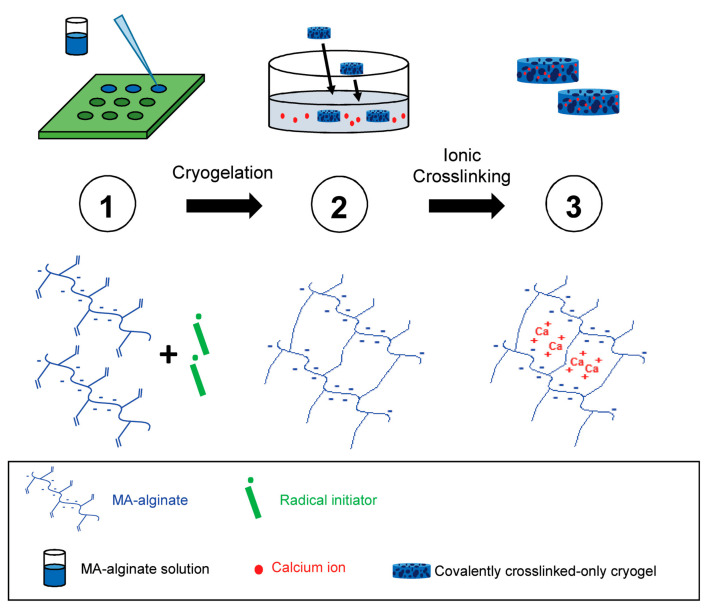
Schematic diagram for the production principle of MA-alginate tough cryogels [7].

Shih et al. produced an injectable tough MA-alginate cryogel as a cancer vaccine. Nowadays, cancer vaccines attract great attention for removing tumor-forming factors and suppressing recurrence of the disease. Previous studies have reported that a covalently crosslinked MA-alginate cryogel vaccine is effective against skin cancer in rodents, but fractured after injection. Based on this study, Shih et al. synthesized a tough cryogel by combining ionic cross-links to covalently crosslinked MA-alginate cryogels by means of calcium ions (Figure 7). In summary, it was investigated whether the obtained MA-alginate cryogels can be injected with a needle without any damage and can be used as a cancer vaccine, and it was observed that it inhibited 80% of tumor formation in the mouse breast cancer model [7].

The 3D bioprinting technique serves as a developing field that allows for the reproduction of human and animal tissues such as skin, nerve tissue, and liver, which are very important in tissue engineering. Béduer et al. developed 3D-printed injectable cryogel scaffolds that could be beneficial for minimally invasive delivery. In the study, it was emphasized that the pore sizes of cryogels created with temperature-controlled 3D printing technology can be adjusted. The characterization of biocompatible and elastic cryogels was performed using techniques such as SEM, injectability, and pore size distribution. In the study conducted with mice, it was concluded that the cryogel scaffolds had very good biocompatibility after three months following their injection [82].

## 5. Conclusions

During the past several decades, supermacroporous cryogels have already utilized a dramatic effect in biomedical applications. As evident from the research done so far, cryogels can be fabricated from diverse precursors, thereby enabling the modulation of its properties that allows for its usage for varied tissues. In this review, we discussed some examples of recent developments in biomedicine applications of injectable cryogels. Studies in recent years are promising for the use and further development of injectable cryogels in the biomedicine field such as diagnosis, cell therapy, drug delivery, and therapeutics. In the area of soft materials processing and refinement, there is still plenty of room for advancement. In the following years, it is thought that the production of more compressible, self-healing, sensitive to external stimuli, and injectable cryogels with macrostructural features will expand their biomedicine use and increase their applicability in different and wider areas.

## Data Availability

Not applicable.

## References

[1] Bencherif S.A., Sands R.W., Bhatta D., Arany P., Verbeke C.S., Edwards D.A., Mooney D.J. (2012). Injectable preformed scaffolds with shape-memory properties. Proc. Nalt. Acad. Sci. USA.

[2] Plieva F.M., Galaev I.Y., Mattiasson B. (2007). Macroporous gels prepared at subzero temperatures as novel materials for chromatography of particulate-containing fluids and cell culture applications. J Sep. Sci..

[3] Mikhalovsky S.V., Savina I.N., Dainiak M., Ivanov A.E., Galaev I.Y., Moo-Young M. (2011). 5.03-Biomaterials/Cryogels, Comprehensive Biotechnology.

[4] Plieva F.M., Galaev I.Y., Noppe W., Mattiasson B. (2008). Cryogel applications in microbiology. Trends Microbiol..

[5] Dainiak M.B., Galaev I.Y., Kumar A., Plieva F.M., Mattiasson B. (2007). Chromatography of living cells using supermacroporous hydrogels, cryogels. Adv. Biochem. Eng. Biotechnol..

[6] Lozinsky V.I. (2018). Cryostructuring of polymeric systems. 50.† cryogels and cryotropic gel-formation: Terms and definitions. Gels.

[7] Shih T.Y., Blacklow S.O., Li A.W., Freedman B.R., Bencherif S., Koshy S.T., Darnell M.C., Mooney D.J. (2018). Injectable, tough alginate cryogels as cancer vaccines. Adv. Healthc. Mater..

[8] Kang H.W., Lee S.J., Ko I.K., Kengla C., Yoo J.J., Atala A. (2016). A 3D bioprinting system to produce human-scale tissue constructs with structural integrity. Nat. Biotechnol..

[9] Dhandayuthapani B., Yoshida Y., Maekawa T., Kumar D.S. (2011). Polymeric scaffolds in tissue engineering application: A review. Int. J. Polym. Sci..

[10] Eggermont L.J., Rogers Z.J., Colombani T., Memic A., Bencherif S.A. (2020). Injectable Cryogels for Biomedical Applications. Trends Biotechnol..

[11] Uzunoğlu G., Çimen D., Bereli N., Çetin K., Denizli A. (2019). Removal of Low-Density Lipoprotein Cholesterol from Blood Plasma via Heparin Modified Cryogels. J. Biomater. Sci. Polym. Ed..

[12] Çimen D., Göktürk I., Yılmaz F. (2016). Removal of Iron by Chelation with Molecularly Imprinted Supermacroporous Cryogel. Artif. Cells Nanomed. Biotechnol..

[13] Çetin K., Denizli A. (2015). 5-Fluorouracil delivery from metal-ion mediated molecularly imprinted cryogel discs. Colloids Surf. B.

[14] Saylan Y., Denizli A. (2019). Supermacroporous Composite Cryogels in Biomedical Applications. Gels.

[15] Plieva F., Huiting X., Galaev I.Y., Bergenståhl B., Mattiasson B. (2006). Macroporous elastic polyacrylamide gels prepared at subzero temperatures: Control of porous structure. J. Mater. Chem..

[16] Okay O., Lozinsky V.I., Okay O. (2014). Synthesis and structure–property relationships of cryogels. Polymeric Cryogels: Macroporous Gels with Remarkable Properties.

[17] Asliyüce S., Bereli N., Uzun L., Onur M.A., Say R., Denizli A. (2010). Ion-imprinted supermacroporous cryogel, for in vitro removal of iron out of human plasma with beta thalassemia. Sep. Purif. Technol..

[18] Çimen D., Denizli A. (2012). Immobilize Metal Affinity Monolithic Cryogels for Cytochrome C Purification. Colloids Surf. B..

[19] Kumari J., Kumar A. (2017). Development of polymer based cryogel matrix for transportation and storage of mammalian cells. Sci. Rep..

[20] Jain E., Karande A.A., Kumar A. (2011). Supermacroporous polymer-based cryogel bioreactor for monoclonal antibody production in continuous culture using hybridoma cells. Biotechnol. Prog..

[21] Carvalho B.M.A., Da Silva S.L., Da Silva L.H.M., Minim V.P.R., Da Silva M.C.H., Carvalho L.M. (2014). Cryogel Poly(acrylamide): Synthesis, Structure and Applications. Sep. Purif. Rev..

[22] Çimen D., Bereli N., Andaç M., Denizli A. (2018). Molecularly imprinted cryogel columns for Concanavalin A purification from jack bean extract. Sep. Sci. Plus..

[23] Plieva F.M., Karlsson M., Aguilar M.R., Gomez D., Mikhalovsky S., Galaev I.Y. (2005). Pore structure in supermacroporous polyacrylamide based cryogels. Soft Matter..

[24] Gun’ko V.M., Savina I.N., Mikhalovsky S.V. (2013). Cryogels: Morphological, structural and adsorption characterisation. Adv. Coll. Int. Sci..

[25] Bereli N., Saylan Y., Uzun L., Say R., Denizli A. (2011). L-Histidine imprinted supermacroporous cryogels for protein recognition. Sep. Purif. Technol..

[26] Inanan T., Tüzmen N., Karipcin F. (2018). Oxime-functionalized cryogel disks for catalase immobilization. Int. J. Biol. Macromol..

[27] Koshy S.T., Ferrante T.C., Lewin S.A., Mooney D.J. (2014). Injectable, Porous, and Cell-Responsive Gelatin Cryogels. Biomaterials.

[28] Singh N.K., Dsouza R.N., Grasselli M., Fernández-Lahore M. (2014). High capacity cryogel-type adsorbents for protein purification. J. Chromatogr. A.

[29] Kuo C.Y., Chen C.H., Hsiao C.Y., Chen J.P. (2015). Incorporation of chitosan in biomimetic gelatin/chondroitin- 6-sulfate/hyaluronan cryogel for cartilage tissue engineering. Carbohydr. Polym..

[30] Baydemir G., Bereli N., Andaç M., Say R., Galaev I.Y., Denizli A. (2009). Supermacroporous poly(hydroxyethyl methacrylate) based cryogel with embedded bilirubin imprinted particles. React. Funct. Polym..

[31] Zhao S., Wang D., Zhu S., Liu X., Zhang H. (2019). 3D cryogel composites as adsorbent for isolation of protein and small molecules. Talanta.

[32] Çimen D., Yılmaz F., Perçin I., Türkmen D., Denizli A. (2015). Dye affinity cryogels for plasmid DNA purification. Mater. Sci. Eng. C.

[33] Wang B.H., Campbell G. (2009). Campbell, G. Formulations of polyvinyl alcohol cryogel that mimic the biomechanical properties of soft tissues in the natural lumbar intervertebral disc. Spine.

[34] O’Brien F.J. (2011). Biomaterials scaffolds for tissue engineering. Mater. Today.

[35] Bakhshpour M., Idil N., Perçin I., Adil Denizli A. (2019). Biomedical Applications of Polymeric Cryogels. Appl. Sci..

[36] Bereli N., Yavuz H., Denizli A. (2020). Protein chromatography by molecular imprinted cryogels. J. Liq Chrom. Relat. Tech..

[37] Lozinsky V.I., Galaev I.Y., Plieva F.M., Savina I.N., Jungvid H., Mattiasson B. (2003). Polymeric cryogels as promising materials of biotechnological interest. Trends Biotechnol..

[38] Hacker M.C., Nawaz H.A. (2015). Multi-functional macromers for hydrogel design in biomedical engineering and regenerative medicine. Int. J. Mol. Sci..

[39] Erdem A., Ngwabebhoh F.A., Yildiz U. (2016). Fabrication and characterization of soft macroporous Jeffamine cryogels as potential materials for tissue applications. RSC Adv..

[40] Konovalova M.V., Markov P.A., Durnev E.A., Kurek D.V., Popov S.V., Varlamov V.P. (2017). Preparation and biocompatibility evaluation of pectin and chitosan cryogels for biomedical application. J. Biomed. Mater. Res..

[41] Savina I.N., Ingavle G.C., Cundy A.B., Mikhalovsky S.V. (2016). A simple method for the production of large volume 3D macroporous hydrogels for advanced biotechnological, medical and environmental applications. Sci. Rep..

[42] Yavuz H., Bereli N., Baydemir G., Andaç M., Türkmen D., Denizli A., Kumar A. (2016). Cryogels: Applications in Extracorporeal Affinity Therapy. Supermacroporous Cryogels: Biomedical and Biotechnological Applications.

[43] Bhat S., Kumar A. (2013). Biomaterials and bioengineering tomorrow’s healthcare. Biomatter..

[44] Cheng L., Ji K., Shih T.Y., Haddad A., Giatsidis G., Mooney D.J., Orgill D.P., Nabzdyk C.S. (2017). Injectable shape-memorizing three-dimensional hyaluronic acid cryogels for skin sculpting and soft tissue reconstruction. Tissue Eng. Part A.

[45] Kim I., Lee S.S., Bae S., Lee H., Hwang N.S. (2018). Heparin functionalized injectable cryogel with rapid shape-recovery property for neovascularization. Biomacromolecules.

[46] Chew S.A., Danti S. (2017). Biomaterial-based implantable devices for cancer therapy. Adv. Healthc. Mater..

[47] Kearney C.J., Mooney D.J. (2013). Macroscale delivery systems for molecular and cellular payloads. Nat. Mater..

[48] Chen M.H., Wang L.L., Chung J.J., Kim Y.H., Atluri P., Burdick J.A. (2017). Methods to assess shear-thinning hydrogels for application as injectable biomaterials. ACS Biomater. Sci. Eng..

[49] Willerth S.M., Sakiyama-Elbert S.E. (2019). Combining stem cells and biomaterial scaffolds for constructing tissues and cell delivery. StemJournal..

[50] Tan H., Marra K.G. (2010). Injectable, biodegradable hydrogels for tissue engineering applications. Materials.

[51] Rezaeeyazdi M., Colombani T., Memic A., Bencherif S.A. (2018). Injectable hyaluronic acid-co-gelatin cryogels for tissue-engineering applications. Materials.

[52] Demir D., Boelgen N. (2017). Synthesis and characterization of injectable chitosan cryogel microsphere scaffolds. Int. J. Polym. Mater. Pol..

[53] Lei K., Tang L. (2019). Surgery-free injectable macroscale biomaterials for local cancer immunotherapy. Biomater. Sci..

[54] Patenaude M., Smeets N.M., Hoare T. (2014). Designing injectable, covalently cross-linked hydrogels for biomedical applications. Macromol. Rapid Commun..

[55] Cloyd J.M., Malhotra N.R., Weng L., Chen W., Mauck R.L., Elliott D.M. (2007). Material Properties in Unconfined Compression of Human Nucleus Pulposus, Injectable Hyaluronic Acid-Based Hydrogels and Tissue Engineering Scaffolds. Eur. Spine J..

[56] Schirmer L., Hoornaert C., Le Blon D., Eigel D., Neto C., Gumbleton M., Welzel P.B., Rosser A.E., Werner C., Ponsaerts P. (2020). Heparin-based, injectable microcarriers for controlled delivery of interleukin-13 to the brain. Biomater. Sci..

[57] Goodarzi K., Shariatzadeh F.J., Solouk A., Akbari S., Mirzadeh H. (2020). Injectable drug loaded gelatin based scaffolds as minimally invasive approach for drug delivery system: CNC/PAMAM nanoparticles. Eur. Polym. J..

[58] Nikolova M.P., Chavali M.S. (2019). Recent advances in biomaterials for 3D scaffolds: A review. Bioact. Mater..

[59] Hixon K.R., Eberlin C.T., Kadakia P.U., McBride-Gagyi S.H., Jain E., Sell S.A. (2018). A comparison of cryogel scaffolds to identify an appropriate structure for promoting bone regeneration. Biomed. Phys. Eng. Exp..

[60] Koshy S.T., Zhang D.K.Y., Grolman J.M., Stafford A.G., Mooney D.J. (2018). Injectable nanocomposite cryogels for versatile protein drug delivery. Acta Biomater..

[61] Bencherif S.A., Sands R.W., Ali O.A., Li W.A., Lewin S.A., Braschler T.M., Shih T.Y., Verbeke C.S., Bhatta D., Dranoff G. (2015). Injectable cryogel-based whole-cell cancer vaccines. Nat. Commun..

[62] Hixon K.R., Lu T., Sell S.A. (2017). A comprehensive review of cryogels and their roles in tissue engineering applications. Acta Biomater..

[63] Cao J., Wang P., Liu Y., Zhu C., Fan D. (2020). Double crosslinked HLC-CCS hydrogel tissue engineering scaffold for skin wound healing. Int. J. Biol. Macromol..

[64] Shiekh P.A., Andrabi S.M., Singh A., Majumder S., Kumar A. (2021). Designing cryogels through cryostructuring of polymeric matrices for biomedical applications. Eur. Polym. J..

[65] Sener G., Hilton S.A., Osmond M.J., Zgheib C., Newsom J.P., Dewberry L., Singh S., Sakthivel T.S., Seal S., Liechty K.W. (2020). Injectable, self-healable zwitterionic cryogels with sustained microRNA-cerium oxide nanoparticle release promote accelerated wound healing. Acta Biomater..

[66] Zhao X., Guo B., Wu H., Liang Y., Ma P.X. (2018). Injectable antibacterial conductive nanocomposite cryogels with rapid shape recovery for noncompressible hemorrhage and wound healing. Nat. Commun..

[67] Çetin K., Aslıyüce S., Idil N., Denizli A. (2020). Preparation of lysozyme loaded gelatin microcryogels and investigation of their antibacterial properties. J. Biomater. Sci. Polym. Ed..

[68] Zhao X., Liang Y., Guo B., Yin Z., Zhu D., Han Y. (2021). Injectable dry cryogels with excellent blood-sucking expansion and blood clotting to cease hemorrhage for lethal deep-wounds, coagulopathy and tissue regeneration. Chem. Eng. J..

[69] Navare K.J., Colombani T., Rezaeeyazdi M., Bassous N., Rana D., Webster T., Memic A., Bencherif S.A. (2020). Needle-injectable microcomposite cryogel scafolds with antimicrobial properties. Sci. Rep..

[70] Larsson E., Boujemaoui A., Malmstrom E., Carlmark A. (2015). Thermoresponsive cryogels reinforced with cellulose nanocrystals. RSC Adv..

[71] Demir D., Özdemir S., Yalçın M.S., Bölgen N. (2020). Chitosan cryogel microspheres decorated with silver nanoparticles as injectable and antimicrobial scaffolds. Int. J. Polym. Mater. Polym. Biomater..

[72] Dimatteo R., Darling N.J., Segura T. (2018). In situ forming injectable hydrogels for drug delivery and wound repair. Adv. Drug Delivery Rev..

[73] Radhouani H., Bicho D., Gonçalves C., Maia F.R., Reis R.L., Oliveira J.M. (2019). Kefiran cryogels as potential scaffolds for drug delivery and tissue engineering applications. Mater. Today Commun..

[74] Memic A., Colombani T., Eggermont L.J., Rezaeeyazdi M., Steingold J., Rogers Z.J., Navare K.J., Mohammed H.S., Bencherif S.A. (2019). Latest advances in cryogel technology for biomedical applications. Adv. Therap..

[75] Zeng Y., Chen C., Liu W., Fu Q., Han Z., Li Y., Feng S., Li X., Qi C., Wu J. (2015). Injectable microcryogels reinforced alginate encapsulation of mesenchymal stromal cells for leak-proof delivery and alleviation of canine disc degeneration. Biomaterials.

[76] Flippova A., Bonini F., Efremova L., Locatelli M., Preynat-Seauve O., Béduer A., Krause K.H., Braschler T. (2021). Neurothreads: Development of supportive carriers for mature dopaminergic neuron differentiation and implantation. Biomaterials.

[77] Bruns J., McBride-Gagyi S., Zustiak S.P. (2018). Injectable and cell-adhesive polyethylene glycol cryogel scaffolds: Independent control of cryogel microstructure and composition. Macromol. Mater. Eng..

[78] Liu W., Li Y., Zeng Y., Zhang X., Wang J., Xie L., Li X., Du Y. (2014). Microcryogels as injecTable 3-D cellular microniches for site-directed and augmented cell delivery. Acta Biomater..

[79] Zeng Y., Zhu L., Han Q., Liu W., Mao X., Li Y., Yu N., Feng S., Fu Q., Wang X. (2015). Preformed gelatin microcryogels as injectable cell carriers for enhanced skin wound healing. Acta Biomater..

[80] Li Y., Yan X., Liu W., Zhou L., You Z., Du Y. (2017). 3D microtissues for injectable regenerative therapy and high-throughput drug screening. J. Vis. Exp..

[81] Villard P., Rezaeeyazdi M., Colombani T., Joshi-Navare K., Rana D., Memic A., Bencherif S.A. (2019). Autoclavable and injectable cryogels for biomedical applications. Adv. Healthc. Mater..

[82] Béduer A., Piacentini N., Aeberli L., Da Silva A., Verheyen C.A., Bonini F., Rochat A., Filippova A., Serex L., Renaud P. (2018). Additive manufacturing of hierarchical injectable scaffolds for tissue engineering. Acta Biomater..

